# Supplementary material for: Relaxation dynamics in bio-colloidal cholesteric liquid crystals confined to cylindrical geometry
Source: Nat Commun. 2020 Sep 15;11:4616. doi: 10.1038/s41467-020-18421-9 (PMC7493995; doi:10.1038/s41467-020-18421-9)
Supplement: Supplementary file 1 — Supplementary Information [file 41467_2020_18421_MOESM1_ESM.pdf]

Supplementary Information for

Relaxation Dynamics in Bio-Colloidal Cholesteric Liquid Crystals Confined to Cylindrical Geometry

Sayyed Ahmad Khadem[1,2], Massimo Bagnani[3], Raffaele Mezzenga[3,4], Alejandro D. Rey[1,2*]

[1]Department of Chemical Engineering, McGill University, Montreal, Quebec H3A 2B2, Canada

[2]Quebec Centre for Advanced Materials, Canada (QCAM/CQMF), Montreal, Quebec, Canada H3A 2K6

[3]Department of Health Sciences and Technology, ETH Zurich, Schmelzbergstrasse 9, Zurich 8092, Switzerland

[4]Department of Materials, ETH Zurich, Wolfgang-Pauli-Strasse 10, Zurich 8093, Switzerland

*To whom correspondence should be addressed. E-mail: alejandro.rey@mcgill.ca

Supplementary Figure 1.

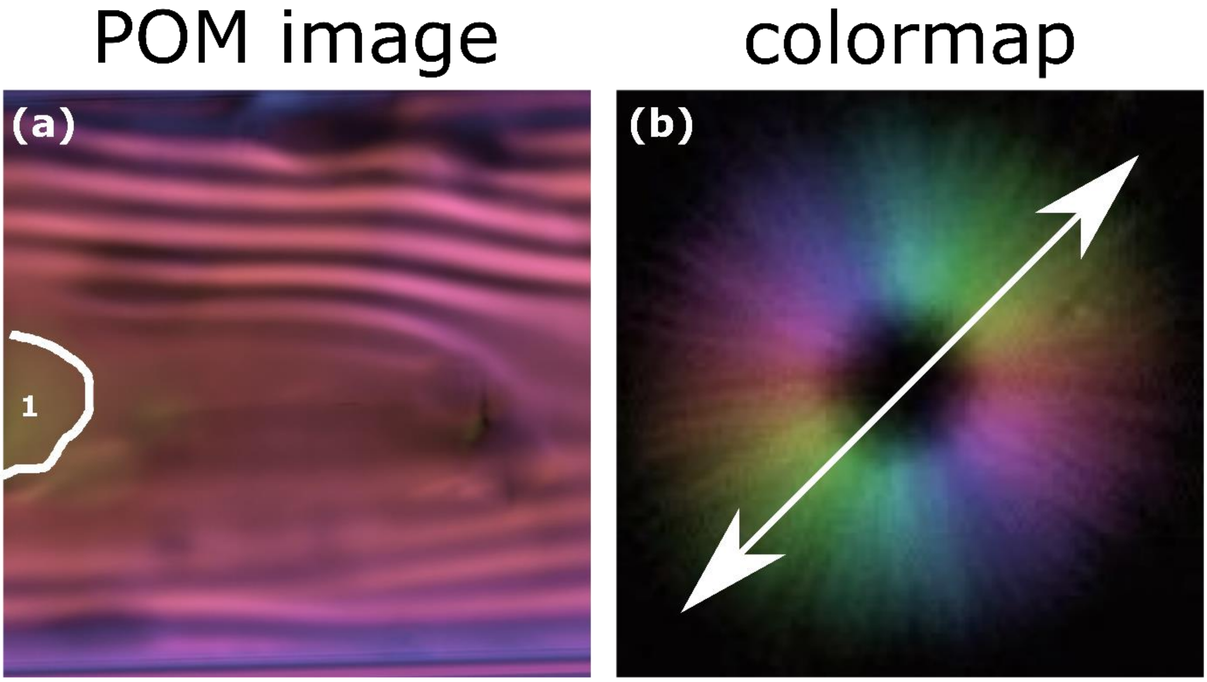

Supplementary Figure 1 | Mapping between microscopy (POM) image and the colormap. (a) Representative microscopy (POM) image, and **(b)** the colormap.

Supplementary Figure 2.

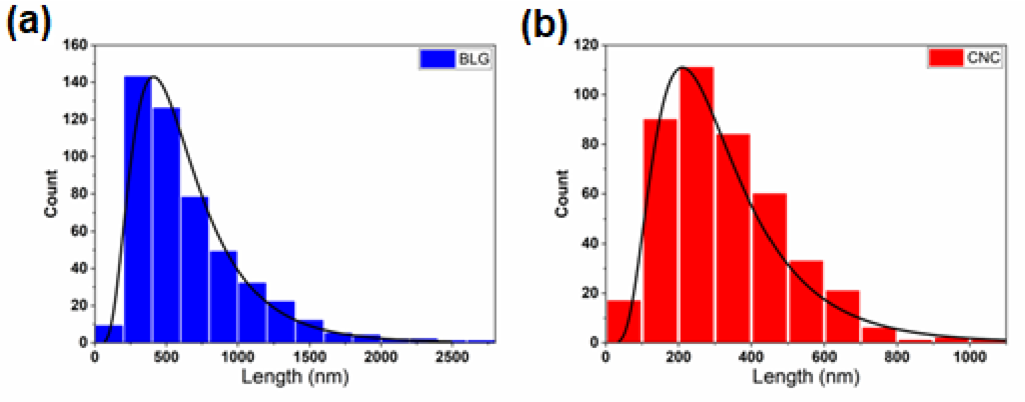

Supplementary Figure 2 | Amyloid and cellulose length distributions. The length distributions of particles extracted from AFM measurements are plotted as histograms and fitted as log-normal distributions (black lines) for BLG amyloid fibrils and CNC in **(a)** and **(b)**, respectively. The average contour length results 652±400 nm and 325±168 nm for BLG and CNC, respectively. The average heights of the fibrils (corresponding to the diameter of the rods) resulted 3.75±0.8 and 4.5±1.0 nm for BLG and CNC, respectively.

Supplementary Figure 3.

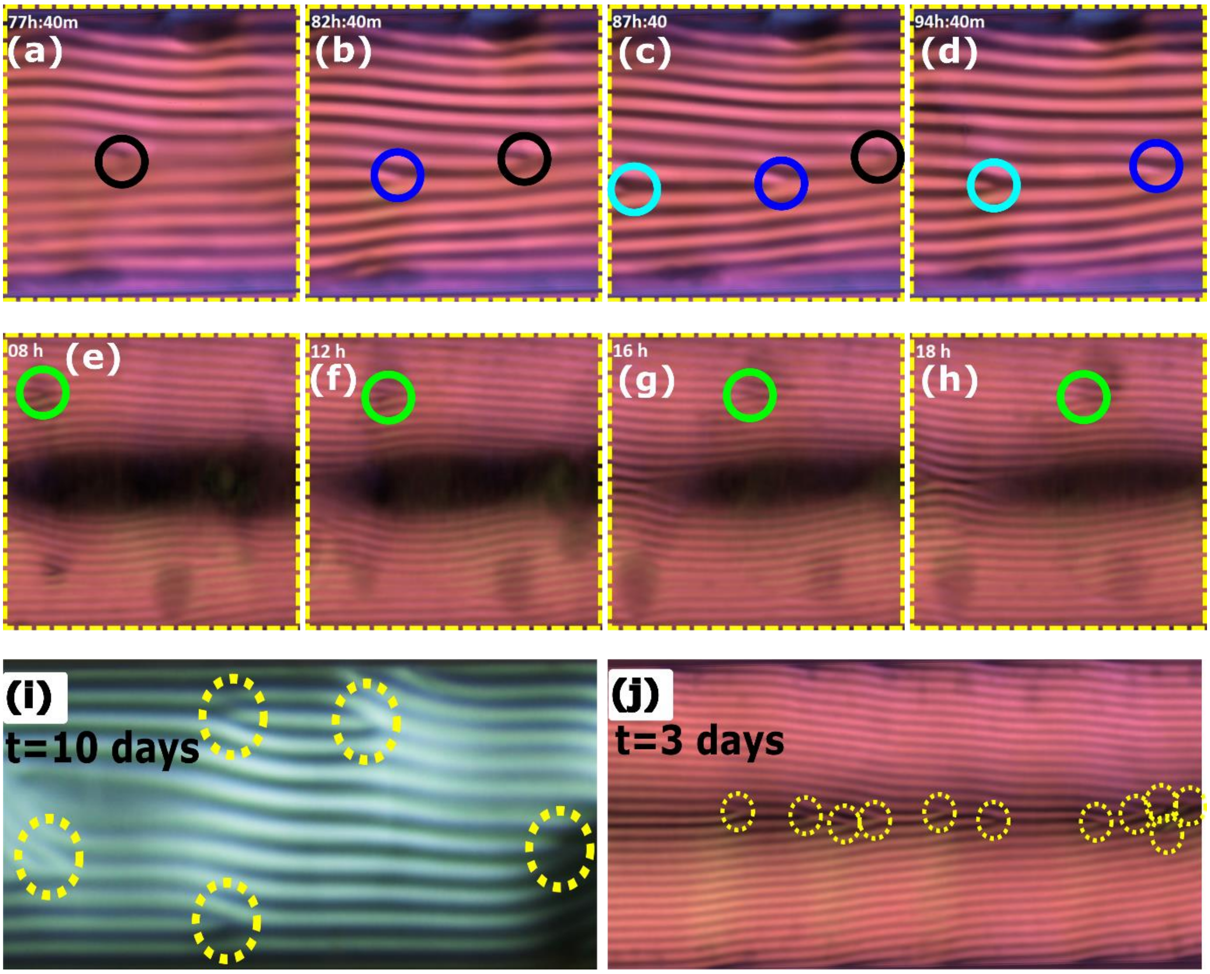

Supplementary Figure 3 | Defects Analysis. The PolScope images depicting the movement of defects for **(a-d)** β-lactoglobulin amyloid fibrils (BLG) and **(e-h)** cellulose nanocrystals (CNC). In the panels (a-h), the circles show defects, and each defect is shown with a unique color. **(i)** The representative POM images of BLG and **(j)** PolScope colormap representation for CNC. In the panels (i, j), the dashed circles show defects; hence, there are 5 defects for BLG and 11 defects for CNC on the larger region (600μm×260μm) after equilibration (10 days for BLG and 3 days for CNC).

Supplementary Figure 4.

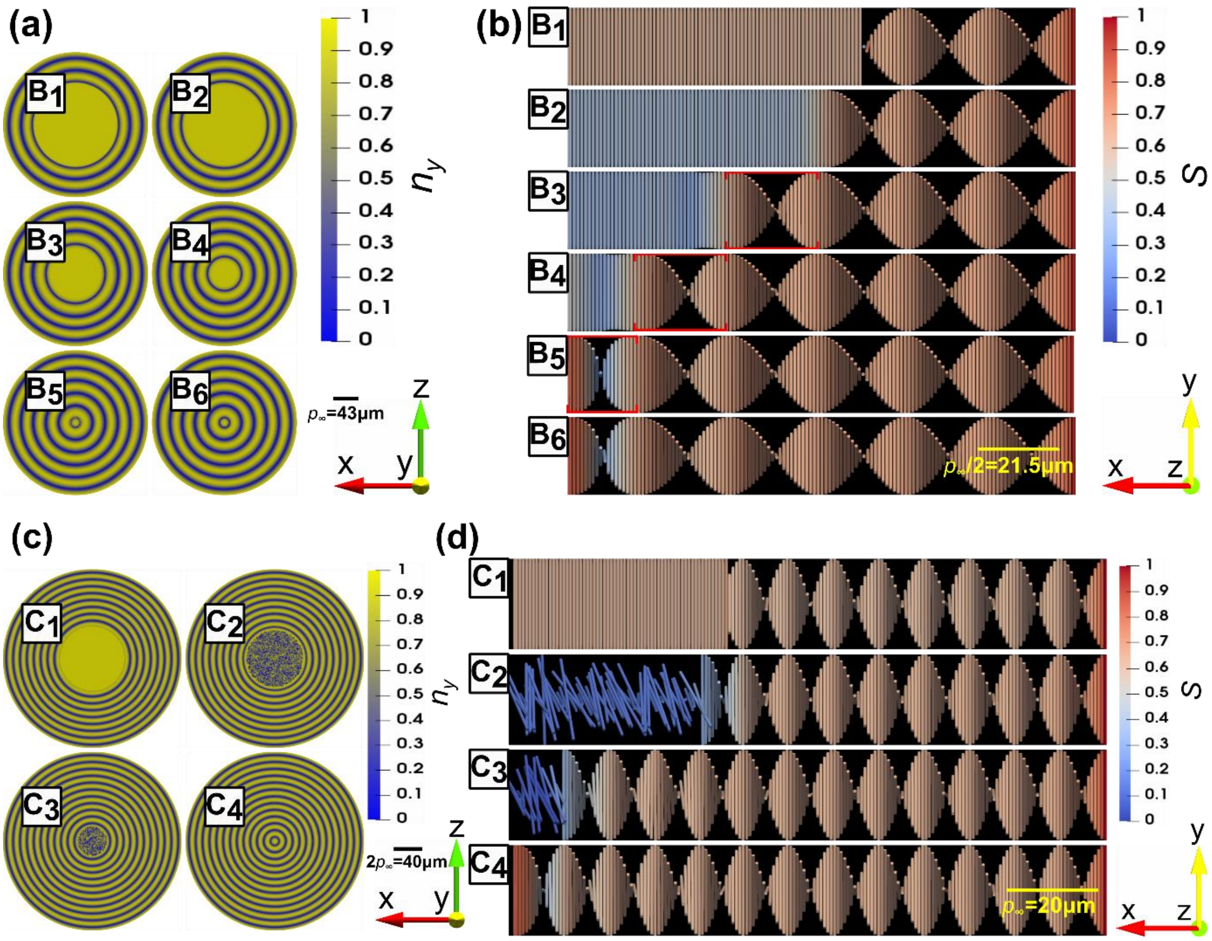

Supplementary Figure 4 | Circular chiral front propagation and fibers configuration along the capillary radius. (a, c) Sequential snapshots of a circular cross-section through the capillary tube demonstrating y-component of the director filed, n_y. Yellow and blue thus exhibit where fibers are parallel and perpendicular to the central axis of the cylinder, respectively. **(b, d)** Fibers orientation along the radius of circular cross-sections depicted in the panels (a, c), respectively. General Note: (a, b) and (c, d) correspond to BLG and CNC, respectively.

Supplementary Figure 5.

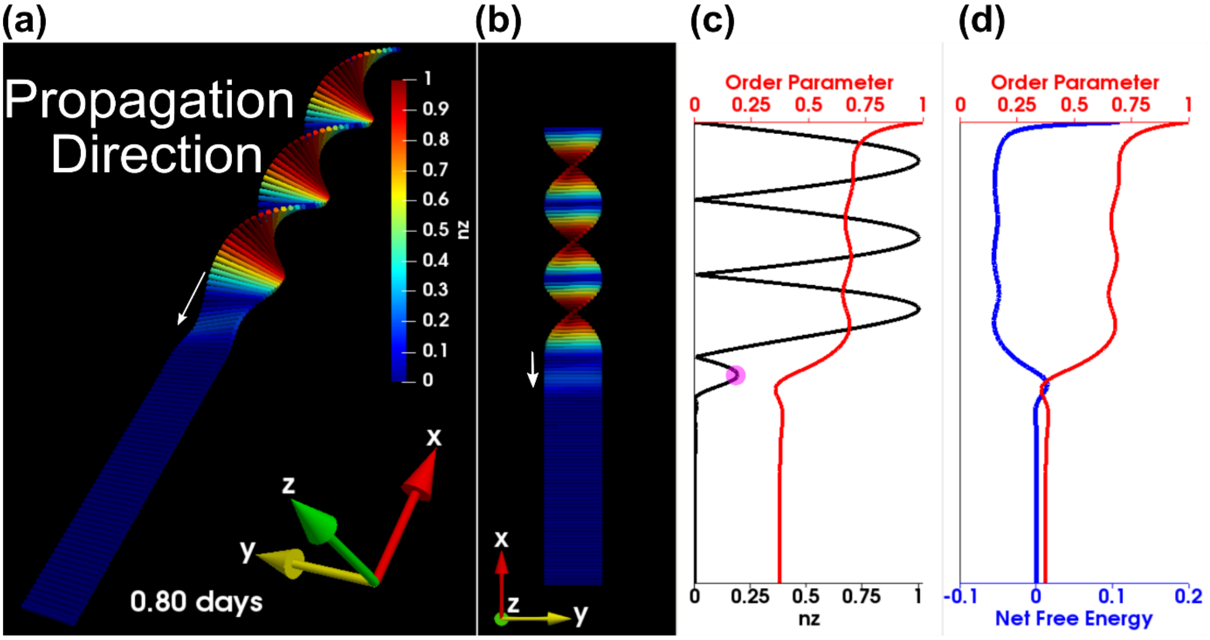

Supplementary Figure 5 | First essential stage in BLG front propagation, *i.e.* Phase(II). (a, b) Fibers configuration along the radius of the capillary cylinder at two different angles of view and color shows z-component of the director field, *i.e.* fiber configuration. **(c)** Quantitative representation of z-component, bottom axis, and order parameter, top axis, along the helix depicted in panel (b). **(d)** Order parameter, top axis, and net free energy, bottom axis, along the helix depicted in (b). Note: y-axes in the panels (c, d) are the length of the helix shown in the panel (b).

Supplementary Figure 6.

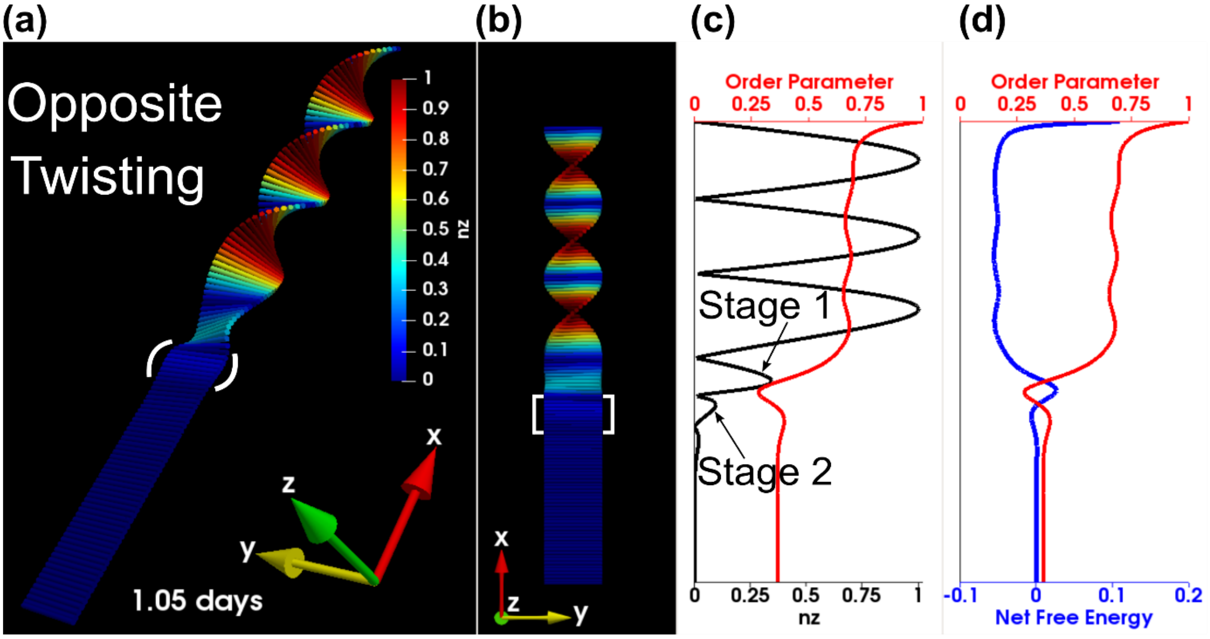

Supplementary Figure 6 | Second essential stage in BLG front propagation, *i.e.* Phase(II). (a, b) Fibers configuration along the radius of the capillary cylinder at two different angles of view and color shows z-component of the director field, *i.e.* fiber configuration. **(c)** Quantitative representation of z-component, bottom axis, and order parameter, top axis, along the helix depicted in panel (b). **(d)** Order parameter, top axis, and net free energy, bottom axis, along the helix depicted in (b). Note: y-axes in the panels (c, d) are the length of the helix shown in the panel (b).

Supplementary Figure 7.

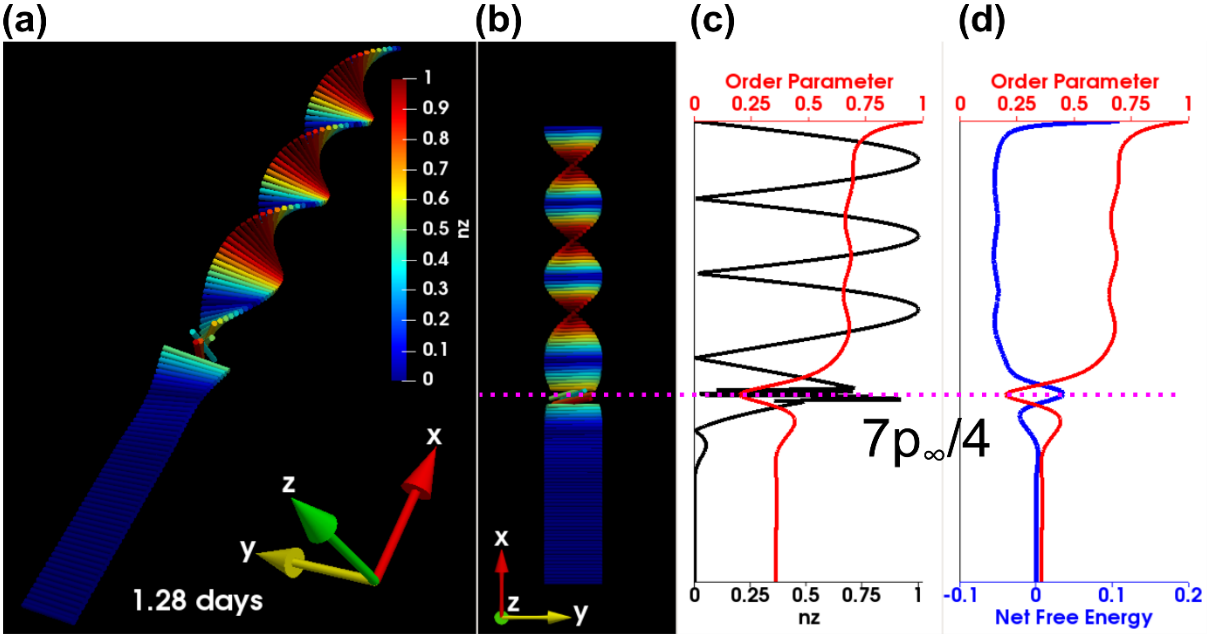

Supplementary Figure 7 | Third essential stage in BLG front propagation, *i.e.* Phase(II). (a, b) Fibers configuration along the radius of the capillary cylinder at two different angles of view and color shows z-component of the director field, *i.e.* fiber configuration. **(c)** Quantitative representation of z-component, bottom axis, and order parameter, top axis, along the helix depicted in panel (b). **(d)** Order parameter, top axis, and net free energy, bottom axis, along the helix depicted in (b). Note: y-axes in the panels (c, d) are the length of the helix shown in the panel (b).

Supplementary Figure 8.

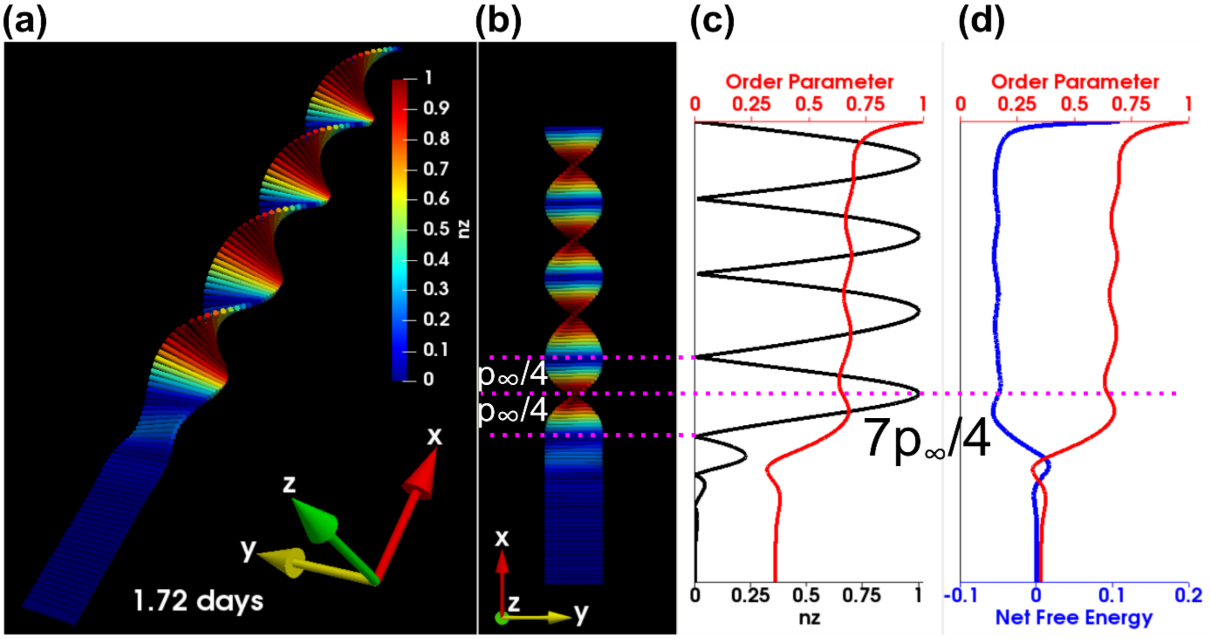

Supplementary Figure 8 | Fourth essential stage in BLG front propagation, *i.e.* Phase(II). (a, b) Fibers configuration along the radius of the capillary cylinder at two different angles of view and color shows z-component of the director field, *i.e.* fiber configuration. **(c)** Quantitative representation of z-component, bottom axis, and order parameter, top axis, along the helix depicted in panel (b). **(d)** Order parameter, top axis, and net free energy, bottom axis, along the helix depicted in (b). Note: y-axes in the panels (c, d) are the length of the helix shown in the panel (b).

Supplementary Figure 9.

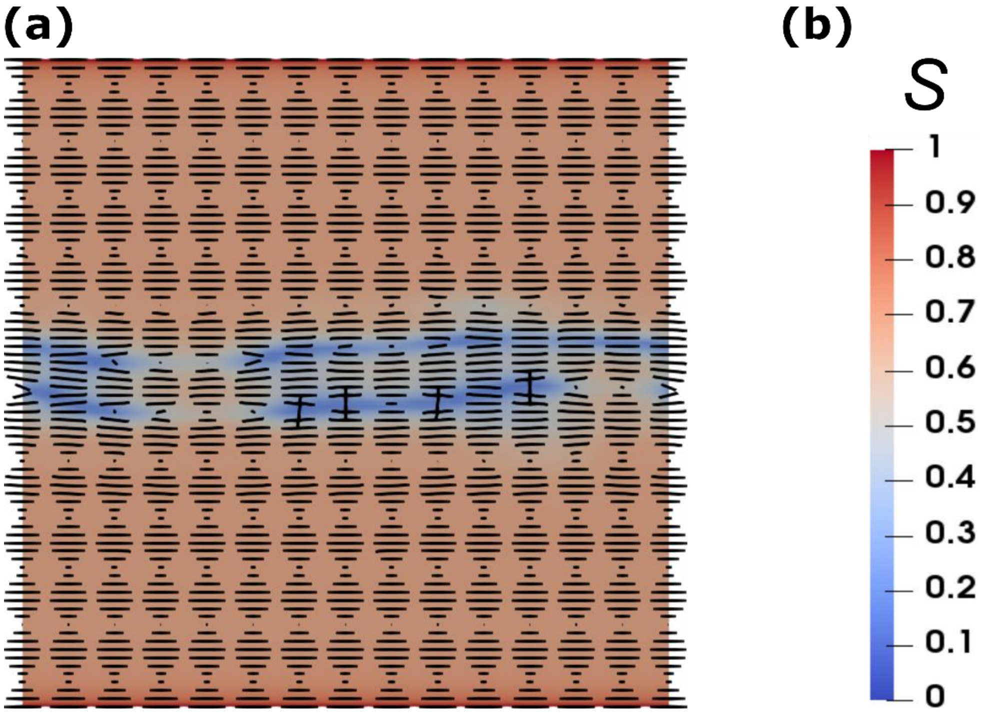

Supplementary Figure 9 | Representative numerical simulation results. (a) Fibers orientation is schematically shown by the director filed and their corresponding order parameter is represented by the blue-to-red spectrum. **(b)** The blue-to-red spectrum.

Supplementary Figure 10.

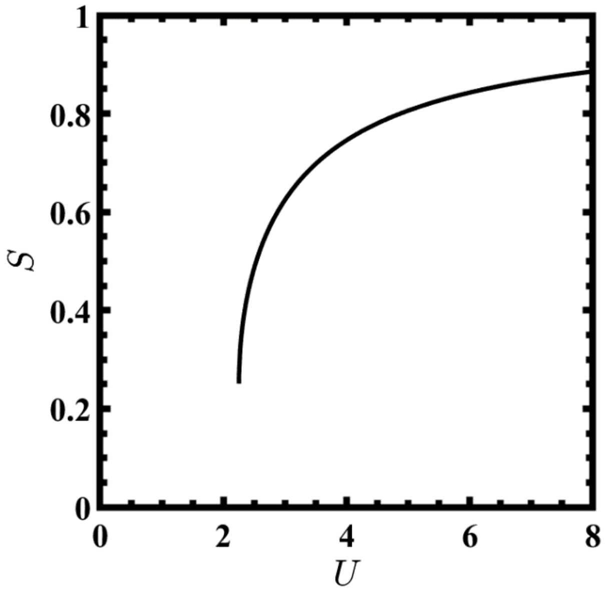

Supplementary Figure 10 | A typical order parameter behavior. The order parameter abruptly drops to zero if the concentration is less than a critical value.

Supplementary Figure 11.

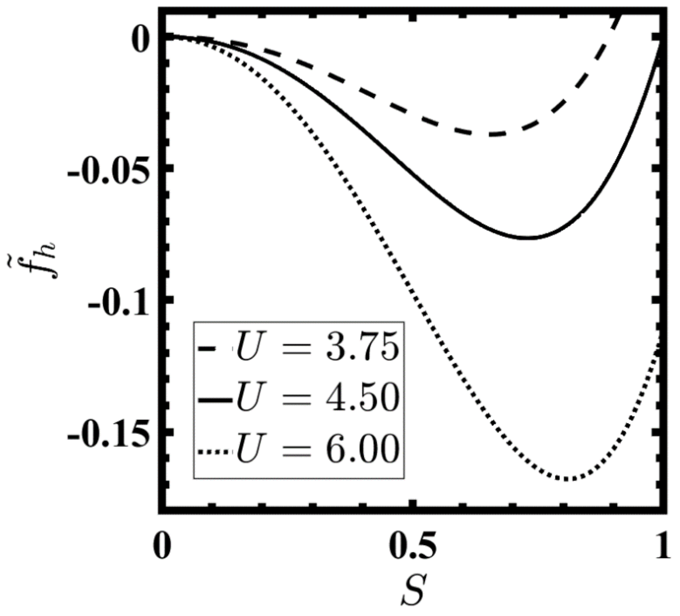

Supplementary Figure 11 | A typical homogenous free energy behavior. Homogenous free energy variation with respect to order parameter at various nematic potentials, $U = 3c/c^*$.

Supplementary Figure 12.

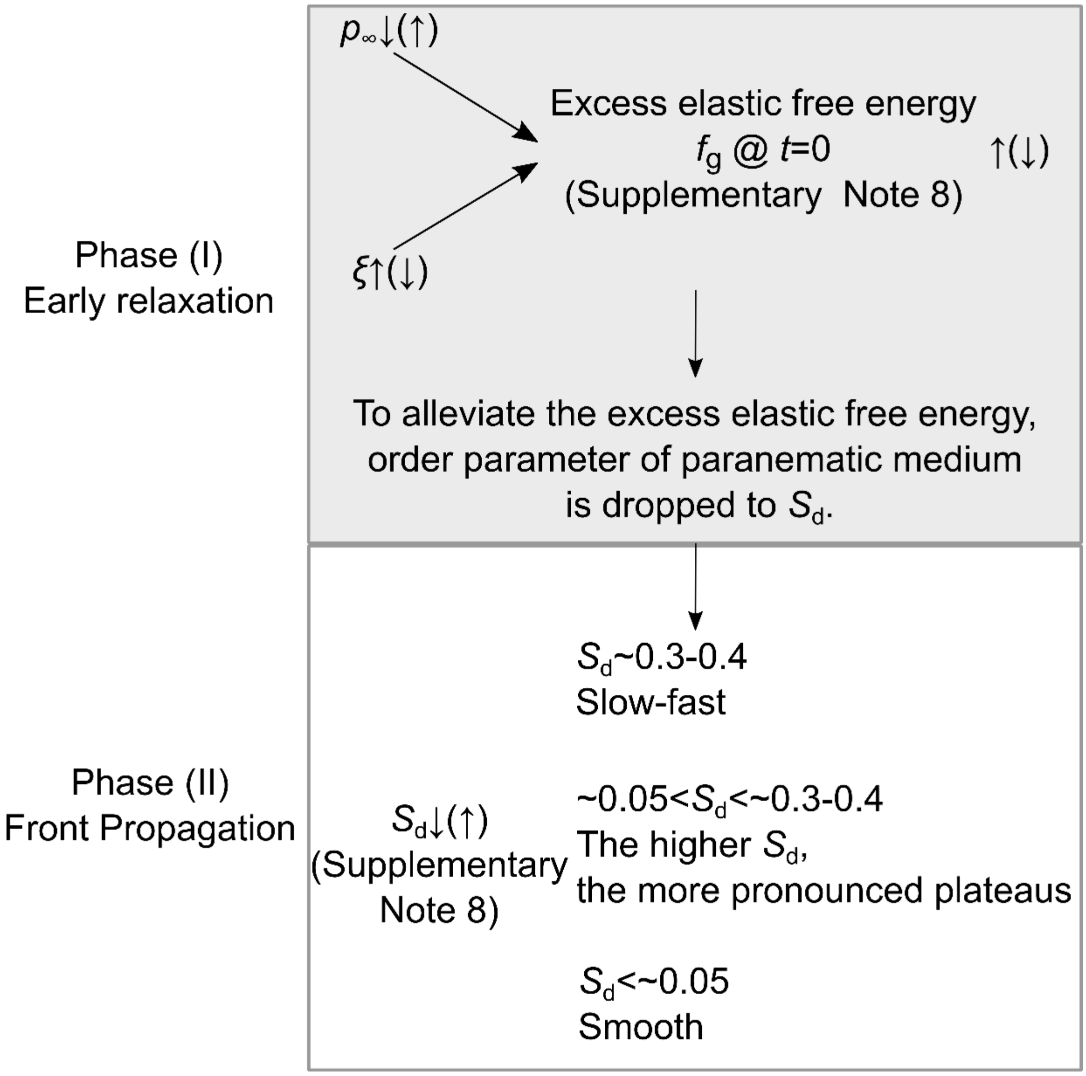

Supplementary Figure 12 | General mechanism governing relaxation dynamics. Summarizing scheme of the impact of coherence length and pitch length on the order parameter of the para-nematic, S_d, and the resulting relaxation dynamics. Upward and downward arrows qualitatively indicate increase and decrease, respectively.

Supplementary Figure 13.

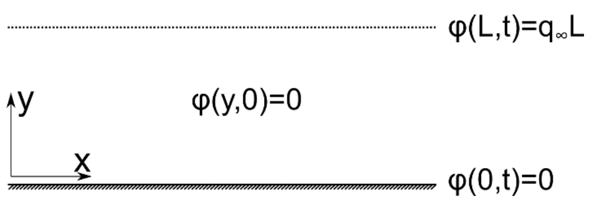

Supplementary Figure 13 | Schematic of an unconfined planar geometry. The bottom line indicates the substrate and the top dash line is the upper boundary condition.

Supplementary Figure 14.

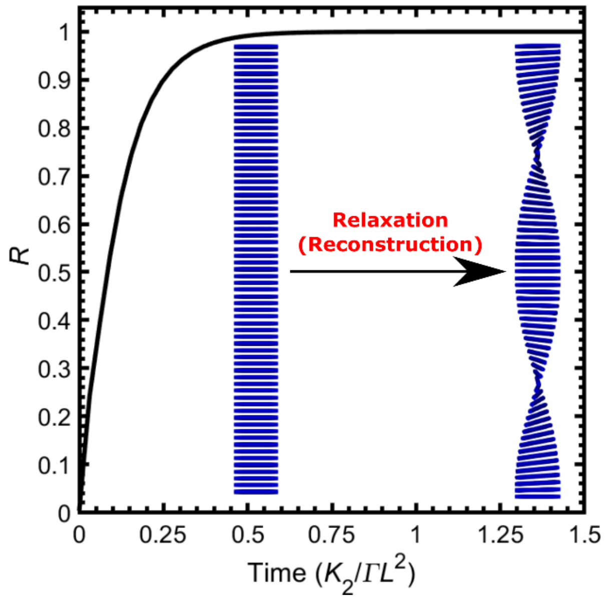

Supplementary Figure 14 | Relaxation dynamics in an unconfined planar geometry. The normalized relaxation progress, *R*, has the first-order dynamic in an unconfined planar geometry.

Supplementary Figure 15.

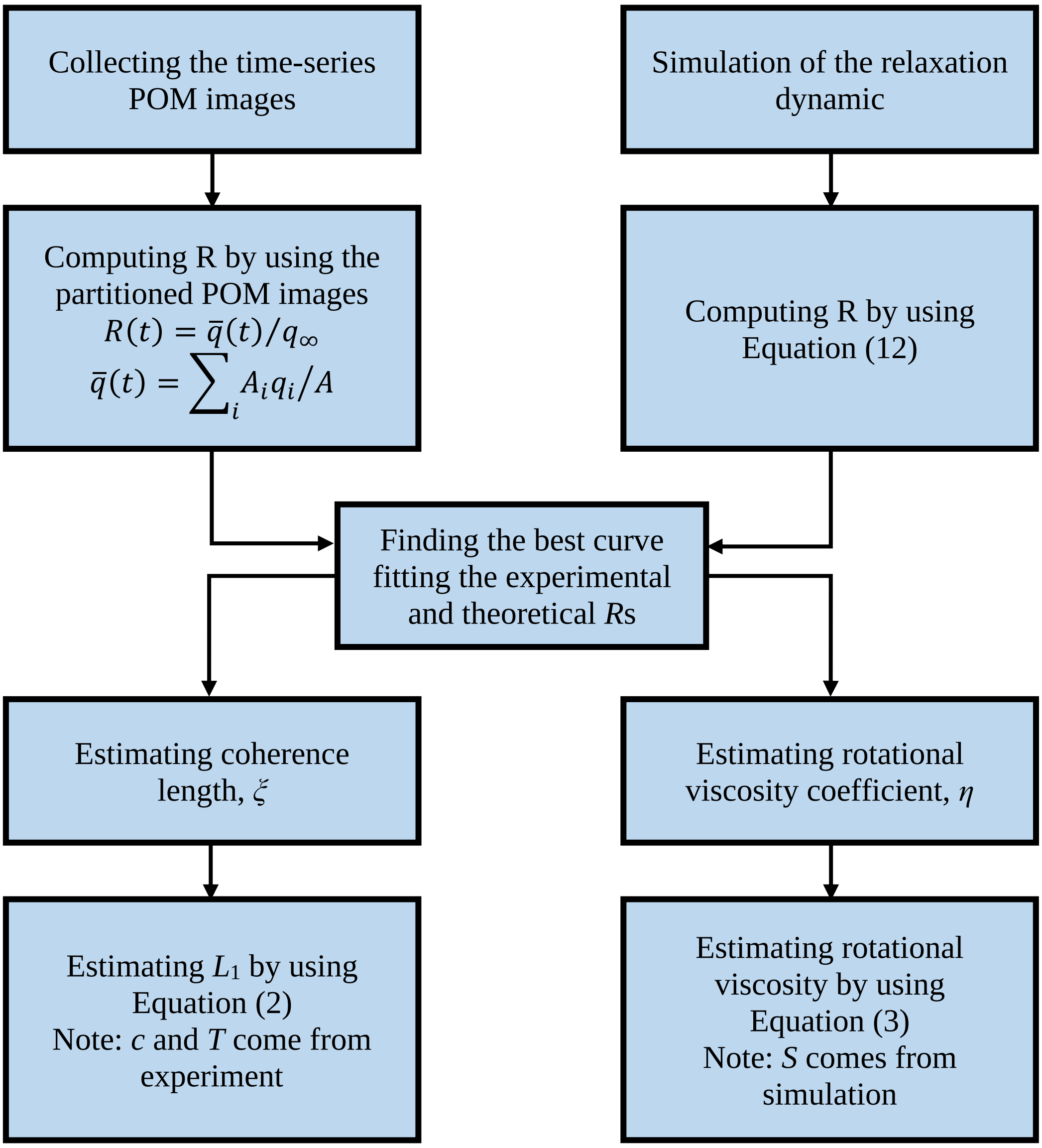

Supplementary Figure 15 | The systematic framework of estimating viscoelastic properties.

Supplementary Figure 16.

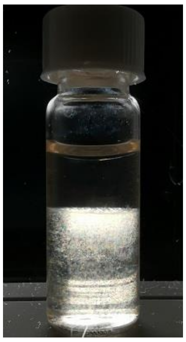

Supplementary Figure 16 | Photograph of phase-separated BLG or CNC dispersion sandwiched between crossed polarizers. From the top to the bottom, phases are Air, Isotropic and Cholesteric, respectively. Note BLG and CNC exactly look like each other in the shown container.

Supplementary Figure 17.

(a)

Simulation Results	Geometry used
Figure 1(m,n)	2D
Figure 2(b, d, f, h, j, l)	2D
Figure 3(b, d, f, h)	2D
Figure 4(a-d)	2D
Supplementary Figure 4	3D
Supplementary Figures 5-8	3D
Supplementary Figure 9	2D
Supplementary Figures 18-20	2D
Supplementary Movie 3	3D
Supplementary Movie 4	3D
Supplementary Movie 5	3D
Supplementary Movie 6	3D

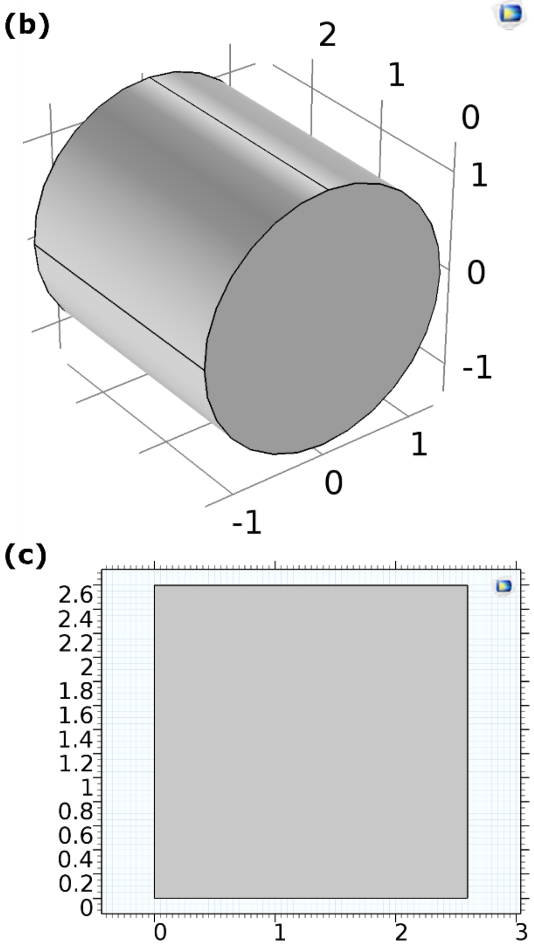

Supplementary Figure 17 | Summary of the geometries used in the present study. (a) The list of Figures and Movies, and the corresponding dimensionalities. **(b)** Representative 3D simulation box. **(c)** Representative 2D simulation box.

Supplementary Figure 18.

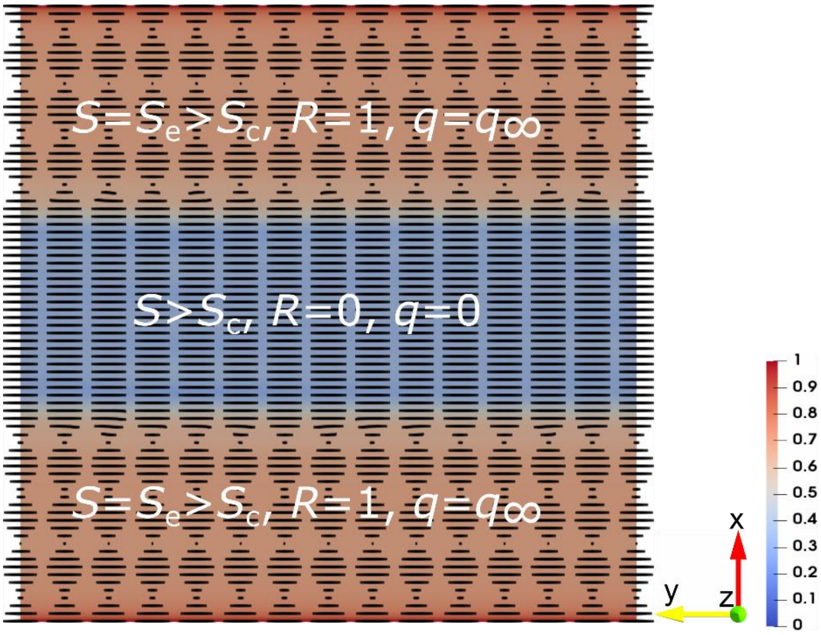

Supplementary Figure 18 | Representative simulation results for BLG. A representative snapshot depicting the BLG relaxation and the corresponding *R*, *q*, *S*, and director field (**n**).

Supplementary Figure 19.

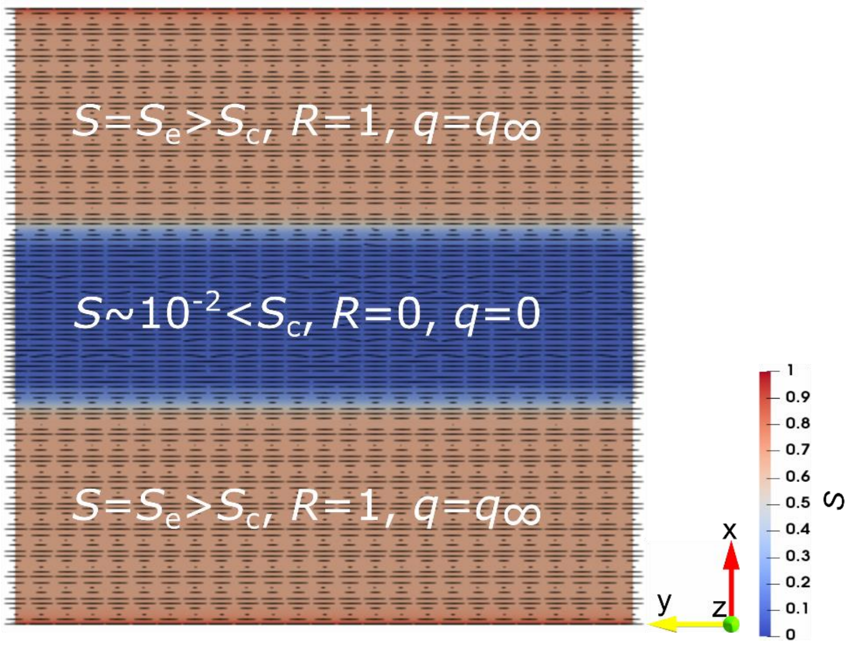

Supplementary Figure 19 | Representative simulation results for CNC. A representative snapshot computed for the CNC relaxation and the corresponding *R*, *q*, *S*, and director field (**n**).

Supplementary Figure 20.

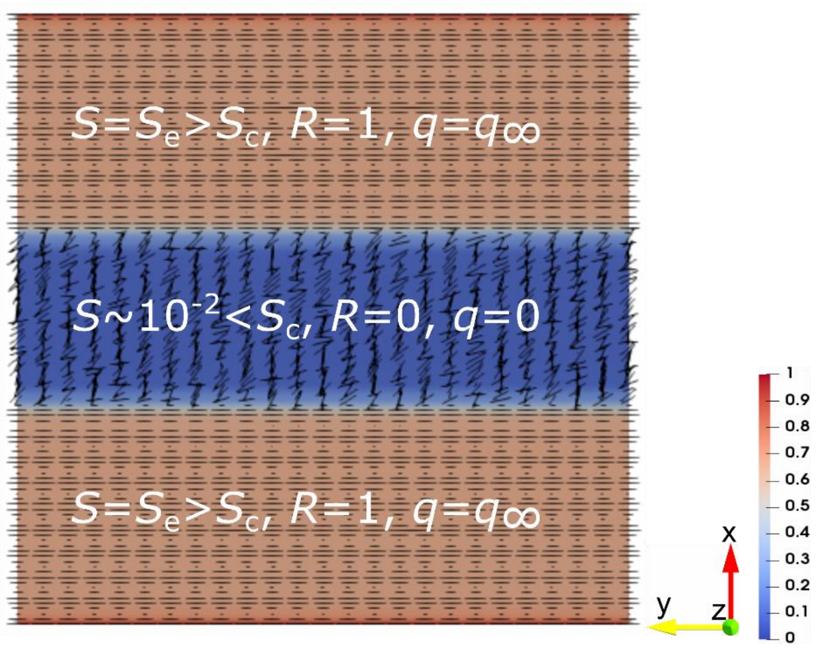

Supplementary Figure 20 | The snapshot visualized for the Supplementary Figure 19.

Supplementary Table 1.

Supplementary Table 1. Impact of parameters on the elastic free energy. Upward and downward arrows qualitatively indicate increase and decrease, respectively.

$p_\infty \downarrow$ and if the phase is para-nematic (*e.g.* in our simulation, the initial phase is para-nematic specified by $\mathbf{n} = \begin{bmatrix} 0 & 1 & 0 \end{bmatrix}$)	$\tilde{f}_{\mathrm{g}} \uparrow$
$S \downarrow$	$\tilde{f}_{\mathrm{g}} \downarrow$
$\xi \downarrow$	$\tilde{f}_{\mathrm{g}} \downarrow$

Supplementary Table 2.

Supplementary Table 2. Parameters used in the present study to capture the dynamics of orientational relaxation.

Parameter	Value
U	4.5
α	19

Supplementary Note 1.

Figures 1 (a-l), Figures 2(c, e, g, i, k), Figures 3(c, e, g), and Figures 4(e-h) depict microscopy (POM) images captured experimentally. The fibers orientation at each point of the POM image can be appreciated by the correspondence between the color of that point and the colormap. For example, region 1 in Supplementary Figure 1**(a)** is approximately between yellow and green. By color mapping with the colormap, we conclude that fibers therein nearly make 45° with the horizontal axis, see the representative arrow shown in the Supplementary Figure 1**(b)**. Similarly, the pink color which is dominant shows the horizontal alignment.

Supplementary Note 2.

The mass fractions, $w_{\mathrm{f}}\left(\%\right)$, were gravimetrically measured as triplicate, giving the same results so there is no error bar available.

The volume densities, $\rho_{\mathrm{f}}\left(\mathrm{g.ml^{-1}}\right)$, were extracted from the literature[1,2].

The length distributions of the rods studied in this paper have been extracted from the atomic force microscopy (AFM) analysis. In particular, 20 µL of 0.01 wt% rods solutions were deposited on freshly cleaved mica for two minutes, rinsed with milliQ water, and scanned with a Multimode VIII scanning probe microscope (Bruker, USA). The images were acquired in tapping mode at ambient condition and the distributions of amyloid fibrils and cellulose nanocrystals (Supplementary Figure 2) were extracted using the open-source code FiberApp[3].

Supplementary Note 3.

We observed that: (1) defects can move throughout the samples, Supplementary Figures 3(a-h), (2) defects may be trapped even after the equilibrium, and (3) number of the trapped defects for CNC is greater than BLG, Supplementary Figures 3(i, j).

Supplementary Note 4.

Supplementary Figures 4(a, c) show that for both BLG and CNC, the chiral front propagates radially, and the ground state domain becomes concentric.

Supplementary Figure 4(b) and Figure 1(m) show that each plateau corresponds to the formation of a half-pitch cholesteric layer; new layers are highlighted by red brackets.

Supplementary Figure 4(d) shows the front propagation of CNC through the para-nematic medium with an extremely low order parameter (*i.e.* isotropic medium).

Supplementary Note 5.

In order to reveal the mechanism of front propagation during Phase (II) for the BLG relaxation, we examined the formation of the cholesteric layers and found that the formation of each half-pitch, $p_\infty/2$, which is equivalent to one sigmoid-like step, obeys four stages (Supplementary Movie 6 reveals the mechanism in full detail).

During Phase (II), the orientational order parameter reduction increases the net free energy since this is largely controlled by the homogeneous free energy, see Figures 4(c, d) and Supplementary Note 8.

Stage (1) in Phase (II): Formation of a weakly chiral layer. During this stage, the chiral front propagates radially inwards replacing the para-nematic phase, see panels (a-c) of Supplementary Figure 5. Across the formed chiral layer, the order parameter decreases and, in turn, the net free energy increases, see panels (c, d) of Supplementary Figure 5. As discussed below, the equilibrium order parameter is $S_e = 0.66$, therefore the configuration of the fibers in Stage 1 is not as robust as that of the ground state and, in consequence, the formed layer is weakly chiral.

Stage (2) in Phase (II): Formation of a weakly chiral layer twisting oppositely. While the weakly chiral front (Stage 1) propagates further, a subtle chiral front emerges with the opposite twisting direction which can be seen by comparison of the panel (a) in Supplementary Figure 5 and Supplementary Figure 6. As a result, there exist two maxima in the z-component of the director field; top and bottom peaks correspond to the formed chiral layers in Stage 1 and Stage 2, respectively. Where these two chiral fronts meet, the order parameter further drops locally which leads to a further increase of net free energy; see panels (c, d) of Supplementary Figure 6. The chirality of Stage 2 is weaker than that of Stage 1 because the thickness and the order

parameter of the chiral layer in Stage 2 are less compared to Stage 1. Therefore, the chirality formed in stage 2 is extremely weak, for this reason, the layer formed in this stage is weakly chiral. We note that this opposite twisting front is a short-lived transient state formed across a few micrometers of the helix and, as can be seen through the Stage 3 and Stage 4, does not affect the overall handedness of cholesteric phase.

Stage (3) in Phase (II): Local order parameter destruction. Fibers located at the intersection of two chiral fronts further lose their order parameter and become essentially isotropic ($S \leq S_c = 0.25$); thereby locally gaining further excess energy. As shown in panels (c, d) of Supplementary Figure 7, loss of order parameter (*i.e.* being isotropic) and energy accumulation at the intersection are reflected as a minimum and a maximum in order parameter and net free energy profiles, respectively. Interestingly, these extrema take place at $7p_\infty/4$ where the orientation of the fibers is supposed to be in azimuthal direction (*i.e.* perpendicular to the central axis of the capillary).

Stage (4) in Phase (II): Simultaneous formation of two $p_\infty/4$ cholesteric layers. The locally stored energy at $7p_\infty/4$, where the order parameter is also minimum and the fibers' configuration is essentially isotropic, is relieved. This excess energy relief then creates two local propagating chiral fronts at $7p_\infty/4$ by which two one-fourth of the pitch, $p_\infty/4$, are formed both inward and outward, see panels (a-c) of Supplementary Figure 8.

Supplementary Figure 8 also corresponds to the beginning of the formation of the next chiral layer. Supplementary Movies 4, 6 confirm that these four stages are periodically repeated until reaching to the ground state.

Supplementary Note 6.

Figures 2(b, d, f, h, j, l) and Figures 3(b, d, f, h) show the order parameter of fibers by use of the blue-to-red spectrum. The blue and red color display low and high order parameter, respectively. Supplementary Figure 9 shows a representative fibers orientation and their order parameter captured by simulation. Note that, throughout our work, the order parameter was obtained by direct numerical simulation.

Supplementary Note 7.

The order-disorder phase transformation is considered as a first-order transition because physical quantities undergo a sharp change through the interface[4]. In this study, we employed the LdG and FOM theories; for this approach, the order parameter has been analytically derived as [5]

$$S=\frac{1}{4}+\frac{1}{4}\sqrt{9-\frac{24}{U}-\frac{96}{U}\pi^2\left(\frac{\xi}{p_\infty}\right)^2} \quad (1)$$

The unique role of the pitch on S was previously discussed [5]. The typical behavior of the order parameter thus looks like Supplementary Figure 10. If concentration does not reach the phase transition threshold, the phase is isotropic and S=0. Upon exceeding the concentration threshold, the liquid-crystalline phase emerges, and S achieves a finite value less than unity. The order parameter at this jump is called the critical order parameter which has been substantially discussed [6-10]. The relatively low critical order parameter in our study is thus $S_c=\frac{1}{4}$.

In this study, $S<S_c$ takes place while the concentration remains at the cholesteric bulk (see section 'Direct numerical simulation').

Supplementary Note 8.

The homogeneous free energy is a polynomial of uniaxial, S, and biaxial, P, order parameters because of[6,11]

$$\mathrm{Tr}\left(\mathbf{Q}^2\right)=\frac{2}{3}\left(S^2+\frac{1}{3}P^2\right) \quad (2)$$

$$\mathrm{Tr}\left(\mathbf{Q}^3\right)=\frac{2}{9}\left(S^3-SP^2\right) \quad (3)$$

In order to understand the behavior of the homogeneous free energy, $\tilde{f}_\mathrm{h}$, with respect to the order parameter, it is reasonable to use the widely-accepted assumption of neglecting the biaxial contribution because the biaxial order parameter is considerably smaller compared to uniaxial order parameter, $P\approx 0$ [7]. Thus, the homogeneous free energy is expressed as

$$\tilde{f}_\mathrm{h}=\frac{1}{3}\left(1-\frac{U}{3}\right)S^2-\frac{2U}{27}S^3+\frac{U}{9}S^4 \quad (4)$$

Supplementary Figure 11 shows that homogenous free energy decreases when the order parameter evolves from a low value to the equilibrium point. Hereafter, for the sake of simplicity, we use the order parameter rather than the uniaxial order parameter throughout the article.

Given the formulation of gradient elastic free energy along with the **Q**-tensor definition, one can see that there is a factor S in all terms.

$$\mathbf{Q} = S\left(\mathbf{nn} - \frac{\boldsymbol{\zeta}}{3}\right) \tag{5}$$

$$\tilde{f}_{\mathrm{g}} = \frac{1}{2}\left(\frac{\xi}{h_0}\right)^2 \left[\left[\tilde{\nabla}\times\mathbf{Q} + 4\pi\left(\frac{h_0}{p_\infty}\right)\mathbf{Q}\right]^2 + a\left[\tilde{\nabla}\cdot\mathbf{Q}\right]^2\right] \tag{6}$$

where $\boldsymbol{\zeta}$ is the 3D unit dyadic. Reduction in the order parameter leads to decrease of the **Q**-tensor, Supplementary Equation (5), in turn, all penalty terms in gradient elasticity, Supplementary Equation (6), become smaller. For simplicity to show the impact of order parameter reduction on the gradient elastic free energy contribution, it is reasonable to assume that the order parameter is independent of space. In this case, the long-range elastic free energy is rewritten as

$$\tilde{f}_{\mathrm{g}} = \frac{1}{2}\left(\frac{\xi}{h_0}\right)^2 S^2 \left[\left[\tilde{\nabla}\times\left(\mathbf{nn} - \frac{\boldsymbol{\zeta}}{3}\right) + 4\pi\left(\frac{h_0}{p_\infty}\right)\left(\mathbf{nn} - \frac{\boldsymbol{\zeta}}{3}\right)\right]^2 + a\left[\tilde{\nabla}\cdot\left(\mathbf{nn} - \frac{\boldsymbol{\zeta}}{3}\right)\right]^2\right] \tag{7}$$

More specifically, the elastic-free energy is weighted by the squared order parameter and the squared coherence length. Therefore, a reduction in the magnitude of the order parameter leads to a lower gradient elastic free energy.

Aside from the impact of order parameter and coherence length on the elastic free energy, the pitch length can also have an impact on elastic free energy, especially affecting the early relaxation. The excess elastic free energy is higher for smaller pitch lengths as the deviation from the ground state increases. Supplementary Table 1 summarizes how elastic free energy is affected by the order parameter, coherence length, and pitch length. It should, however, be mentioned that we did not apply the simplifying assumption of the constant order parameter in direct numeric simulations.

Supplementary Note 9.

In the given confinement, the relaxation dynamics depend on pitch length and coherence length. In this section, we focus on how a relaxation dynamic gradually switches from a slow-fast relaxation dynamic to a smooth relaxation dynamic and vice versa.

The impact of pitch length, coherence length, and order parameter on the elastic free energy are summarized in Supplementary Table 1. Interaction between these parameters determines the type of relaxation dynamics, see Supplementary Figure 12. Depending on material-related properties of pitch length and coherence length, an excess elastic free energy is induced at the beginning of relaxation as a result of the non-equilibrium configuration of fibers (*i.e.* para-nematic phase). As explained in the Supplementary Note 8 and shown in Supplementary Table 1, Supplementary Figure 12, the excess elastic free energy is positively and inversely affected by the coherence length and pitch length, respectively. The next step in the relaxation is to relieve excess energy by reducing the order parameter. This step takes place to cancel out, or at least to alleviate the initial excess energy. So far Phase (I) is completed. Phase (II) is chiral front propagation. Using direct numerical simulations, we found that there can be three cases for type of relaxation dynamics as illustrated in Supplementary Figure 12.

Supplementary Note 10.

The purpose of this section is to show that confinement, *i.e.* curvature of the capillary, has a considerable impact on relaxation dynamics. The well-established Frank-Oseen (FO) free energy can also describe the relaxation dynamics of a single helix[12].

$$f_{\mathrm{FO}} = \frac{1}{2}K_1\left(\nabla\cdot\mathbf{n}\right)^2 + \frac{1}{2}K_2\left(\mathbf{n}\cdot\left(\nabla\times\mathbf{n}\right)+q_\infty\right)^2 + \frac{1}{2}K_3\left(\mathbf{n}\times\left(\nabla\times\mathbf{n}\right)\right)\cdot\left(\mathbf{n}\times\left(\nabla\times\mathbf{n}\right)\right) \tag{8}$$

K_1, K_2, K_3 stand for splay, twist and bend modes of deformation, respectively. $\mathbf{n}$ is unit vector representing the fibers' orientation. Before proceeding to reveal the curvature role in relaxation dynamics by making use of Frank-Oseen, it should be mentioned that Frank-Oseen is a subset of the **Q**-tensor Frank-Oseen-Mermin gradient energy, Equation (9). These two methods, FO and FOM, can be converted to each other by use of **Q**-tensor definition, Supplementary Equation (5), and mapping between L_i and K_i discussed in references[12,13].

To meet the constraint of unit length, the unit vector is considered as $\mathbf{n}=\left[\sin\left(\varphi\left(y,t\right)\right) \quad 0 \quad \cos\left(\varphi\left(y,t\right)\right)\right]$ where $\varphi\left(y,t\right)$ is the angle between the director and x-axis. This consideration simplifies the elasticity free energy as

$$f_{\mathrm{FO}} = \frac{1}{2}K_2\left(q_\infty - \frac{\partial\varphi(y,t)}{\partial y}\right)^2 \tag{9}$$

By use of Supplementary Equation (9), the Leslie–Ericksen model reduces to

$$\frac{\partial\varphi}{\partial t} = \frac{K_2}{\Gamma}\frac{\partial^2\varphi}{\partial y^2} \tag{10}$$

where Γ is rotational viscosity of a single helix. Given Supplementary Figure 13, the initial and boundary conditions are expressed by

$$\text{IC: } \varphi(y,0) = 0 \tag{11}$$

$$\text{BC:}\begin{cases} \varphi(0,t) = 0 \\ \varphi(L,t) = q_\infty L \end{cases} \tag{12}$$

Therefore, the analytical solution of relaxation dynamics reads

$$\varphi(y,t) = q_\infty y + \sum_{k=1}^{\infty} A_k \sin\left(\frac{k\pi}{L}y\right)\exp\left(-\frac{K_2}{\Gamma}\left(\frac{k\pi}{L}\right)^2 t\right) \tag{13}$$

$$A_k = 2(-1)^k Lq_\infty / k\pi \tag{14}$$

Note that the upper boundary condition comes from the steady solution, $\varphi(L) = q_\infty L$. Knowing the steady-state solution yields the instantaneous chiral wavevector definition as $q(y,t) = \varphi(y,t)/y$ which meets both boundaries of relaxation (*i.e.* $q(y,0) = 0$ and $q(y,\infty) = q_\infty$). Therefore, the relaxation progress, *R*, is expressed as

$$R = \frac{\bar{q}(t)}{q_\infty} = 1 + \sum_{k=1}^{\infty}\left(\frac{2(-1)^k}{k\pi}\right)\left(\int_0^L \sin\left(\frac{k\pi}{L}\zeta\right)\Big/\zeta \,\mathrm{d}\zeta\right)\exp\left(-\frac{K_2}{\Gamma}\left(\frac{k\pi}{L}\right)^2 t\right) \tag{15}$$

The Supplementary Figure 14 illustrates that relaxation generally obeys the first-order dynamic in an unconfined planar geometry. Thus, slow-fast relaxation does not emerge for the unconfined planar system.

In conclusion, relaxation dynamics also depend on the curvature. Characterizing the curvature impact on relaxation is beyond the scope of this work.

Supplementary Note 11.

As can be seen in Supplementary Figure 15, the best curves fitting the experiment and simulation result in estimation of rotational viscosity coefficient, η, and coherence length, ξ. Then, L_1 and rotational viscosity are estimated by use of Equation (2) and Equation (3), respectively.

Supplementary Note 12.

The capillaries were filled with the birefringent solution which is the bulk cholesteric. According to the thermodynamics of phase equilibria, the bulk cholesteric is at a constant concentration equal to the upper binodal curve, see Supplementary Figure 16.

Supplementary Note 13.

The finite element (FE) technique with biquadratic basis functions was employed to carry out the simulations. In this regard, the governing equations, Equations (5-9), along with axillary conditions, Equations (10-11), and parameters tabulated in Supplementary Table 2 were implemented in the General PDE solver of COMSOL Multiphysics 5.3a on our in-house supercomputer. Note that the model used in this study is a tensorial equation, leading to five second-order time-dependent nonlinear coupled PDEs. Time stepping was executed using Backward Differentiation Formula (BDF) with varying orders from one (known as the backward Euler method) to five.

All simulations are performed in a 3D computational domain except those which are intended for comparison with the POM images. Supplementary Figure 17 shows the geometries and the simulation type used in this study.

Supplementary Note 14.

The dimensionless normalized relaxation progress curve, R, is computed in the lateral plane (xy-plane), see Figure 1(o). The ideal director field representing a monodomain along with x-axis reads

$$\mathbf{n} = \left[\sin(q_\infty z) \quad -\cos(q_\infty z) \quad 0\right] \tag{16}$$

Moreover, we have $\mathbf{Q}:\nabla\times\mathbf{Q} = S^2\mathbf{nn}:\nabla\times\mathbf{nn}$ —this identity is proven in accordance with **Q**-tensor definition and tensorial operations, explained as follows.

The **Q**-tensor is defined as $\mathbf{Q} = S\left(\mathbf{nn} - \boldsymbol{\delta}/3\right)$ in which $\boldsymbol{\delta}$ denotes the second rank unit dyadic. Substitution of **Q**-tensor definition in $\mathbf{Q}:\nabla\times\mathbf{Q}$ yields

$$\mathbf{Q}:\nabla\times\mathbf{Q} = S\left(\mathbf{nn} - \boldsymbol{\delta}/3\right):\nabla\times\mathbf{Q} = S\mathbf{nn}:\nabla\times\mathbf{Q} - S\,\boldsymbol{\delta}:\nabla\times\mathbf{Q}/3 \tag{17}$$

As $\boldsymbol{\delta}:\nabla\times\mathbf{Q} = \mathrm{Tr}(\nabla\times\mathbf{Q})$ and $\mathrm{Tr}(\nabla\times\mathbf{Q}) = 0$ due to the symmetric traceless property of the **Q**-tensor, Supplementary Equation (17) reduces to

$$\mathbf{Q}:\nabla\times\mathbf{Q} = S\mathbf{nn}:\nabla\times\mathbf{Q} \tag{18}$$

Once again using the **Q**-tensor definition to find the equivalent form of the curl of **Q**-tensor, $\nabla\times\mathbf{Q} = S\nabla\times\mathbf{nn}$, and Supplementary Equation (18) finally gives

$$\mathbf{Q}:\nabla\times\mathbf{Q} = S^2\mathbf{nn}:\nabla\times\mathbf{nn} \tag{19}$$

Having substituted Supplementary Equation (16) into Supplementary Equation (19), the relation between **Q**-tensor and the relaxed chiral wavevector becomes $q_\infty = -\mathbf{Q}:\nabla\times\mathbf{Q}/S^2$. In addition, knowing the fact that $\mathbf{Q}:\mathbf{Q} = 2S^2/3$ leads to

$$q_\infty = -\frac{2}{3}\frac{\mathbf{Q}:\nabla\times\mathbf{Q}}{\mathbf{Q}:\mathbf{Q}} \tag{20}$$

Because the quantity on the right-hand side approaches to the definite value of the relaxed chiral wavevector in the course of time, in order to quantify the relaxation, we define the dimensionless spatio-temporal chiral wavevector as

$$\tilde{q}(\tilde{\mathbf{x}},\tilde{t}) = -\frac{2}{3}\frac{\mathbf{Q}:\tilde{\nabla}\times\mathbf{Q}}{\mathbf{Q}:\mathbf{Q}} \tag{21}$$

Supplementary Equation (21) satisfactorily meets the limits of relaxation because it is simplified to $\tilde{q}(\tilde{\mathbf{x}},\tilde{t})=0$ (equivalently $\tilde{p}_\infty(\tilde{\mathbf{x}},\tilde{t}) = \infty$) and $\tilde{q}(\tilde{\mathbf{x}},\tilde{t}) = \tilde{q}_\infty = 2\pi/\tilde{p}_\infty$ for achiral and chiral nematic phases, respectively. The tilde symbol denotes dimensionless quantities (*e.g.* $\tilde{\nabla} = h_0\nabla$). Hereafter, we omit the tilde for brevity.

Owing to fact that Supplementary Equation (21) is derived for the ideal director field represented in Supplementary Equation (16), and knowing that $\bar{q}(t)$ is computed by direct numerical simulation, there could be a slight difference in the prediction of q_∞ at equilibrium. In this regard, we re-define the chiral wavevector as $q(\mathbf{x},t) = -\chi\mathbf{Q}:\nabla\times\mathbf{Q}/\mathbf{Q}:\mathbf{Q}$ and therefore

$$\bar{q}(t) = -\frac{\chi}{A_t}\int_A \frac{\mathbf{Q}:\nabla\times\mathbf{Q}}{\mathbf{Q}:\mathbf{Q}}\mathrm{d}A \tag{22}$$

where A_t stand for the total area of the lateral plane (xy-plane). χ plays a scaling role in order to reach q_∞. χ is quite close to the ideal value that is 0.67, see Supplementary Equation (21). For example, $\chi_{\mathrm{BLG}} = 0.63$ and $\chi_{\mathrm{CNC}} = 0.59$. The exact value of χ can also be formulated by the use of the relaxation boundary condition that $q_\infty = -\frac{\chi}{A_t}\left(\int_A \frac{\mathbf{Q}:\nabla\times\mathbf{Q}}{\mathbf{Q}:\mathbf{Q}}\mathrm{d}A\right)_{\mathrm{e}}$ in which subscript e shows the equilibrium point. Therefore, the relaxation progress, $R(t) = \bar{q}(t)/q_\infty$, is expressed by

$$R(t) = \frac{\int_A \frac{\mathbf{Q}:\nabla\times\mathbf{Q}}{\mathbf{Q}:\mathbf{Q}}\mathrm{d}A}{\left(\int_A \frac{\mathbf{Q}:\nabla\times\mathbf{Q}}{\mathbf{Q}:\mathbf{Q}}\mathrm{d}A\right)_{\mathrm{e}}} \tag{23}$$

Now that the normalized relaxation progress, Supplementary Equation (23) or Equation (12), is formulated and our experimental-theoretical approach reveals that the dark zone in the POM images is a para-nematic phase with the order parameter of nearly 0 to 0.4, the conceptual understanding of this quantity (*R*) deserves more discussion. As explained in the paper, when *R* is computed *via* the discretization of the time-series POM images, *R* is taken to be 0 and 1 in dark and fingerprint partitions, respectively. *R*=0 is equivalent to *q*=0 signifying that the phase is para-nematic and *R*=1 indicates $q=q_\infty$ representing that the phase is cholesteric. In the case of BLG relaxation (*i.e.* Figure 2), the *R* distribution theoretically becomes as what is shown in Supplementary Figure 18.

In the case of CNC, the para-nematic phase loses its order parameter to an extremely low value, S~10^{-2}, see Supplementary Figure 19.

The actual fibers' orientation can be understood in light of two factors. First, the uniaxial director field, **n**, representing the average fibers' orientation. Second, the uniaxial order parameter, *S*, describing the strength of fibers alignment around **n**. Fibers lie perfectly parallel to **n** if *S*=1 and the resulting phase becomes more crystal-like. In the case of $S \approx S_c$~0.7, fibers

retain both fluidity and crystallinity (orientational order), corresponding to the liquid-crystalline phase. Finally, $S<S_c$ indicates that the actual fibers' orientation can be less aligned around **n**; hence, the phase possesses more fluid-like characteristics rather than crystalline ones[5,8,10,14-16]. As explained in the Supplementary Note 7, there is no unanimous agreement on the S_c value; however, S_c=0.25 suits for the theory used in our study. Accordingly, wherever $S<S_c$, the fibers' orientation is randomly visualized in order to emphasize the concept of critical order parameter and the fact that orientational ordering is weak, see Supplementary Figure 20.

Wherever $S<S_c$, the phase can also be called isotropic due to the fact that the correlation existing among fibers is insignificant. However, the distinguishing point that should be taken into account is that the concentration of the isotropic phase is still at the upper binodal curve, which is unequivocally greater than the critical order-disorder transition, see "Direct numerical simulation" section for discussion on the concentration field in the present study.

Supplementary Note 15.

The purpose of this note is to show that the **Q**-tensor spatio-temporal evolution equation can be projected into the vector force balance equation containing the divergence of the total stress tensor. The spatio-temporal orientational ordering is expressed by

$$\gamma \frac{\partial \mathbf{Q}}{\partial t} = -\left(\frac{\partial f}{\partial \mathbf{Q}}\right)^{[s]} + \left(\nabla \cdot \left(\frac{\partial f}{\partial \nabla \mathbf{Q}}\right)\right)^{[s]} = \mathbf{H}^{[s]} \tag{24}$$

where f is the Helmholtz free energy density per unit volume and **H** is the molecular field. To derive the 3D force balance equation, the double inner product of Supplementary Equation (24) and $\nabla\mathbf{Q}$ yields

$$\gamma(\nabla\mathbf{Q}):\left(\frac{\partial \mathbf{Q}}{\partial t}\right) = -(\nabla\mathbf{Q}):\left\{\left(\frac{\partial f}{\partial \mathbf{Q}}\right)^{[s]} + \left(\nabla \cdot \left(\frac{\partial f}{\partial \nabla \mathbf{Q}}\right)\right)^{[s]}\right\} \tag{25}$$

Knowing that **Q** is symmetric traceless leads to dropping [s], and using the transpose of third-order tensors, we rewrite Supplementary Equation (25) as:

$$\gamma\left(\frac{\partial \mathbf{Q}}{\partial t}\right):(\nabla\mathbf{Q})^T = -\left(\frac{\partial f}{\partial \mathbf{Q}}\right):(\nabla\mathbf{Q}\ )^T + \left(\nabla \cdot \left(\frac{\partial f}{\partial \nabla \mathbf{Q}}\right)\right):(\nabla\mathbf{Q})^T = \mathbf{H}:(\nabla\mathbf{Q})^T \tag{26}$$

Given that f can be considered as homogenous and gradient contributions, we then formulate the gradient of f.

$$f = f\left(\mathbf{Q},\nabla\mathbf{Q}\right) = f_h\left(\mathbf{Q}\right) + f_g\left(\mathbf{Q},\nabla\mathbf{Q}\right) \tag{27}$$

$$\nabla f = \frac{\partial f}{\partial \mathbf{Q}} : \left(\nabla\mathbf{Q}\right)^T + \frac{\partial f}{\partial \nabla\mathbf{Q}} \vdots \left(\nabla\nabla\mathbf{Q}\right)^T \tag{28}$$

Now we focus on the last term $\frac{\partial f}{\partial \nabla\mathbf{Q}} \vdots \left(\nabla\nabla\mathbf{Q}\right)^T$ which can be written as $\frac{\partial f}{\partial \nabla_p \mathbf{Q}_{mo}} \vdots \nabla_i \nabla_p \mathbf{Q}_{mo}$ by making use of a dual index notation method (Einstein and indicial free) to facilitate the subsequent tensor derivations:

$$\frac{\partial f}{\partial \nabla_p \mathbf{Q}_{mo}} \vdots \nabla_i \nabla_p \mathbf{Q}_{mo} = \frac{\partial f}{\partial \nabla_p \mathbf{Q}_{mo}} \vdots \nabla_p \nabla_i \mathbf{Q}_{mo} = \nabla_p \cdot \left\{ \frac{\partial f}{\partial \nabla_p \mathbf{Q}_{mo}} : \nabla_i \mathbf{Q}_{mo} \right\} - \left(\nabla_p \cdot \left(\frac{\partial f}{\partial \nabla_p \mathbf{Q}_{mo}} \right) \right) : \nabla_i \mathbf{Q}_{mo} \tag{29}$$

Or equivalently

$$\frac{\partial f}{\partial \nabla\mathbf{Q}} \vdots \left(\nabla\nabla\mathbf{Q}\right)^T = \nabla \cdot \left\{ \frac{\partial f}{\partial \nabla\mathbf{Q}} : \left(\nabla\mathbf{Q}\right)^T \right\} - \left(\nabla \cdot \left(\frac{\partial f}{\partial \nabla\mathbf{Q}} \right) \right) : \left(\nabla\mathbf{Q}\right)^T \tag{30}$$

Substituting Supplementary Equation (30) into Supplementary Equation (28) yields

$$-\nabla f + \nabla \cdot \left\{ \frac{\partial f}{\partial \nabla\mathbf{Q}} : \left(\nabla\mathbf{Q}\right)^T \right\} = -\frac{\partial f}{\partial \mathbf{Q}} : \left(\nabla\mathbf{Q}\right)^T + \left(\nabla \cdot \left(\frac{\partial f}{\partial \nabla\mathbf{Q}} \right) \right) : \left(\nabla\mathbf{Q}\right)^T \tag{31}$$

Comparing Supplementary Equations (26, 31) results in

$$\gamma \left(\frac{\partial \mathbf{Q}}{\partial t} \right) : \left(\nabla\mathbf{Q}\right)^T = -\nabla f + \nabla \cdot \left\{ \frac{\partial f}{\partial \nabla\mathbf{Q}} : \left(\nabla\mathbf{Q}\right)^T \right\} = \mathbf{H} : \left(\nabla\mathbf{Q}\right)^T \tag{32}$$

Additionally, we know that the mechanical bulk total elastic stress tensor **T** is

$$\mathbf{T} = \mathrm{f}\,\mathbf{I} + \mathbf{T}^E \tag{33}$$

$$\mathbf{T}^{\mathrm{E}} = -\frac{\partial f_g}{\partial \nabla\mathbf{Q}} : \left(\nabla\mathbf{Q}\right)^T \tag{34}$$

where $\mathbf{T}^{\mathrm{E}}$ is the Ericksen stress tensor, and the pressure p=-f is minus the free energy density. Then, the bulk vector force balance Supplementary Equation (25) reads:

$$-\gamma(\nabla\mathbf{Q}):\left(\frac{\partial\mathbf{Q}}{\partial t}\right)=\nabla\cdot\mathbf{T} \tag{35}$$

The total stress tensor, Supplementary Equations (33, 34), carries information on f and $-\frac{\partial f_g}{\partial\nabla\mathbf{Q}}:(\nabla\mathbf{Q})^T$. The vector mechanical force balance, Supplementary Equation (35), carries less information than the original tensorial **Q**-tensor equation, Supplementary Equation (24) since the former is a vector equation and the latter a tensor equation. Consequently, when we simulate the **Q**-tensor process in the absence of mass velocity (**v**=0), the **Q**-tensor model carries more information by just focusing on the tensorial variational derivative of the free energy instead of the vectorial divergence of the stress.

The total elastic stress **T** is always present $\mathbf{T}\neq\mathbf{0}$ and, in consequence, the elastic force **F** which is the divergence of the total bulk elastic stress is also nonzero, $\mathbf{F}=\nabla\cdot\mathbf{T}\neq\mathbf{0}$ as **F** is balanced by $\gamma\left(\frac{\partial\mathbf{Q}}{\partial t}\right):(\nabla\mathbf{Q})^T+\mathbf{F}=\mathbf{0}$. To sum up, there is no applied external stress in this experiment or in this model, and there is only internal stress (**T**) properly embedded in the modeling used in this study.

Supplementary References

1 Nyström, G., Arcari, M. & Mezzenga, R. Confinement-induced liquid crystalline transitions in amyloid fibril cholesteric tactoids. *Nat. Nanotech.* **13**, 330-336, (2018).

2 Nyström, G., Arcari, M., Adamcik, J., Usov, I. & Mezzenga, R. Nanocellulose fragmentation mechanisms and inversion of chirality from the single particle to the cholesteric phase. *ACS nano* **12**, 5141-5148 (2018).

3 Usov, I. & Mezzenga, R. FiberApp: an open-source software for tracking and analyzing polymers, filaments, biomacromolecules, and fibrous objects. *Macromolecules* **48**, 1269-1280 (2015).

4 Gobeaux *et al*. Cooperative Ordering of Collagen Triple Helices in the Dense State. *Langmuir* **23**, 6411-6417, (2007).

5 De Luca, G. & Rey, A. D. Chiral front propagation in liquid-crystalline materials: Formation of the planar monodomain twisted plywood architecture of biological fibrous composites. *Phys. Rev.E,* **69**, 011706, (2004).

6 Khadem, S. A. & Rey, A. D. Thermodynamic Modelling of Acidic Collagenous Solutions: From Free Energy Contributions to Phase Diagrams. *Soft Matter,* (2019).

7 Khadem, S. A. & Rey, A. D. Theoretical Platform for Liquid-Crystalline Self-Assembly of Collagen-Based Biomaterials. *Front. Phy.*, **7**, (2019).

8 Matsuyamaa, A. & Hirashima, R. Phase separations in liquid crystal-colloid mixtures. *Jour. Chem. Phys.* **128**, 11, (2008).

9 Das, S. K. & Rey, A. D. Computational modelling of multi-phase equilibria of mesogenic mixtures. *Comp. Mat. Sci.* **29**, 152-164, (2004).

10 Doi, M. & Edwards, S. F. *The theory of polymer dynamics*. Vol. 73 (1988).

11 Gennes, P. G. d. & Prost, J. *The Physics of Liquid Crystals*. (1995).

12 Muševič, I. *Liquid Crystal Colloids*. (2017).

13 Zhang, R., Zhou, Y., Rahimi, M. & De Pablo, J. J. Dynamic structure of active nematic shells. *Nat. Commun.* **7**, 13483 (2016).

14 De Luca, G. & Rey, A. D. Ringlike cores of cylindrically confined nematic point defects. *Jour. Chem. Phy.* **126**, 094907 (2007).

15 Gupta, G. & Rey, A. D. Texture rules for concentrated filled nematics. *Phys. Rev. Lett.* **95**, 127802 (2005).

16 De Luca, G. & Rey, A. D. Point and ring defects in nematics under capillary confinement. *Jour. Chem. Phy.* **127**, 104902 (2007).
