# Supplementary material for: Relaxation dynamics in bio-colloidal cholesteric liquid crystals confined to cylindrical geometry
Source: Nat Commun. 2020 Sep 15;11:4616. doi: 10.1038/s41467-020-18421-9 (PMC7493995; doi:10.1038/s41467-020-18421-9)
Supplement: Supplementary file 3 — Description of Additional Supplementary Files [file 41467_2020_18421_MOESM3_ESM.pdf]

Description of Additional Supplementary Files

File Name: Supplementary Movie 1. Click Here to Watch

Description: The experimental time-series POM images showing relaxation of BLG for one week.

File Name: Supplementary Movie 2. Click Here to Watch

Description: The experimental time-series POM images showing relaxation of CNC for one day.

File Name: Supplementary Movie 3. Click Here to Watch

Description: The blue-to-yellow spectrum indicates the y-component of the director field (fibers configuration) in an arbitrary circular cross-section of the cylindrical capillary. Yellow and blue thus correspond to fibers aligned parallel and perpendicular to the central axis of the cylinder (y-axis), respectively.

Fiber orientation along the diameter parallel to the z-axis is superposed onto the cross-section. Additionally, fibers are colored in accordance with their order parameters; the blue-to-red spectrum indicates the order parameter extremes 0 to 1, respectively.

File Name: Supplementary Movie 4. Click Here to Watch

Description: Supplementary Movie 4 consists of four panels described as follows.

Panel (I): Fiber configuration along the diameter parallel x-axis. Note that the fibers' configuration along any arbitrary diameter has identical behavior.

Panel (II): Half of the helix shown in Panel (I) in order that the relaxation can readily be visible.

Panel (III): Order parameter (top axis) and z-component of director field (bottom axis) along the radius (helix) illustrated in Panel (II).

Panel (IV): Order parameter (top axis) and net free energy (bottom axis) along the radius (helix) illustrated in Panel (II).

File Name: Supplementary Movie 5. Click Here to Watch

Description: The description of panels is the same as Supplementary Movie 4. Like the slow-fast mechanism, here there are two phases. Phase (I) in which the order parameter of the para-nematic medium drops from S_{i}=0.6 to $S_{\mathrm{d}} \approx 0$, to decrease excess elastic free energy. In fact, the para-nematic state turns isotropic during Phase (I). Phase (II) is only chiral front propagation invading the isotropic medium.

File Name: Supplementary Movie 6. Click Here to Watch

Description: The purpose of Supplementary Movie 6 is to reveal the mechanism of cholesteric layers formation. Supplementary Movie 6 consists of four panels described as follows.

Panel (I, II): Fibers' configuration along the radius parallel to the x-axis of the cylindrical capillary at two different view angles. The color map shows the magnitude of the z-component of the director field (*i.e.* fiber alignment). For example, blue represents fibers aligned parallel to the central axis of the cylinder(y-axis) and thus their z-components are zero. Red indicates fibers aligned perpendicular to the central axis of the cylinder(y-axis) and are along the z-axis. Note that the fibers' configuration along any arbitrary radius has identical behavior.

Panel (III): Order parameter (top axis) and z-component of director field (bottom axis) along the radius(helix) illustrated in Panel (II).

Panel (IV): Order parameter (top axis) and net free energy (bottom axis) along the radius(helix) illustrated in Panel (II
